# Systematic Approximations to Susceptible-Infectious-Susceptible Dynamics on Networks

**DOI:** 10.1371/journal.pcbi.1005296

**Published:** 2016-12-20

**Authors:** Matt J. Keeling, Thomas House, Alison J. Cooper, Lorenzo Pellis

**Affiliations:** 1 Zeeman Institute: SBIDER, University of Warwick, Coventry, United Kingdom; 2 Mathematics Institute, University of Warwick, Coventry, United Kingdom; 3 School of Life Sciences, University of Warwick, Coventry, United Kingdom; 4 School of Mathematics, University of Manchester, Manchester, United Kingdom; 5 School of Engineering, University of Warwick, Coventry, United Kingdom; Duke University, UNITED STATES

## Abstract

Network-based infectious disease models have been highly effective in elucidating the role of contact structure in the spread of infection. As such, pair- and neighbourhood-based approximation models have played a key role in linking findings from network simulations to standard (random-mixing) results. Recently, for SIR-type infections (that produce one epidemic in a closed population) on locally tree-like networks, these approximations have been shown to be exact. However, network models are ideally suited for Sexually Transmitted Infections (STIs) due to the greater level of detail available for sexual contact networks, and these diseases often possess SIS-type dynamics. Here, we consider the accuracy of three systematic approximations that can be applied to arbitrary disease dynamics, including SIS behaviour. We focus in particular on low degree networks, in which the small number of neighbours causes build-up of local correlations between the state of adjacent nodes that are challenging to capture. By examining how and when these approximation models converge to simulation results, we generate insights into the role of network structure in the infection dynamics of SIS-type infections.

## Author Summary

Networks are now widely used to model infectious diseases, but have posed significant mathematical challenges. Recently analytic results have been obtained for ‘one-off’ network epidemics that follow the SIR paradigm, but these results do not carry over to other scenarios—most significantly to many sexually transmitted infections, where accounting for network structure is vital. Here, we show that it is possible to obtain the large-population dynamics of such diseases on networks through systematic approximations. We focus on a mathematically challenging case of SIS dynamics on networks with low degree.

## Introduction

There is a strong and deep connection between networks and the spread of infectious diseases [1–9]. Virtually all infections can be thought of as propagating through a network of (epidemiologically-relevant) contacts between individuals in the population, with the structure of this underlying network determining much of the infection dynamics. Therefore an understanding of population-level transmission at the scale of individual hosts is closely linked to a study of the properties of the underlying transmission network. Recent advances in network science have highlighted how both local and global structure of the network are key in the dynamics of infection [2, 10–13].

While networks are being increasingly used for airborne and close-contact infections (such as influenza [14] and RSV [15]) which spread through social contacts, the epidemiological network literature was originally formulated for sexually transmitted infections (STIs) where the network is generally more clearly defined. Classic examples include homosexual contact networks from early HIV studies [1] and the Colorado-Springs study of sexual contacts in the high-risk heterosexual population [16]. While a focus on STIs has substantial advantages in terms of determining the network, it also places constraints on the epidemiological dynamics that need to be considered. The overwhelming majority of STIs (e.g. chlamydia or gonorrhoea, although not HIV) can be approximated using the Susceptible-Infected-Susceptible (SIS) paradigm, where infected individuals are treated and recover to the susceptible state, and hence are able to be re-infected multiple times. Although SIS models are inherently lower-dimensional than their SIR (Susceptible-Infected-Recovered) counterparts, potential reinfection of the same individual (multiple times) leads to more complex dynamical behaviour between neighbouring nodes on a network and makes it more difficult to generate tractable results [17].

When details of the complete network are available, and we are dealing with a particular applied problem, then the most straightforward approach is to simulate the dynamics of infection on the given network (e.g. [5, 16, 18]). However in anything but ideal circumstances simulation may be problematic. For example, using simulations alone: it may be difficult to understand sensitivity to elements in network structure or biases in the way the network connections were sampled; it is computationally challenging to infer epidemiological parameters; and it may be difficult to gain a robust understanding of the causal determinants of the observed dynamics. Approximations that maintain the analytic tractability of traditional ODE (Ordinary Differential Equation) models, but take account of elements of network structure provide a possible solution. These have been quite successful for ‘one off’ epidemics that obey the SIR paradigm, with notable advances including the ‘effective degree’ approach of Ball and Neal [19], the probability-generating function approach of Volz [20] (and its reduction to a single dynamical equation by Miller [21]) and the model of Lindquist et al. [22] (originally also called ‘effective-degree’ model, but to which we refer throughout as ‘neighbourhood model’ to avoid confusion with the earlier use of this terminology). Such models, together with pairwise or related approximations [23, 24] discussed extensively in this paper, have been shown to be exact methods of calculation of marginal probabilities for the stochastic SIR model for finite explicitly known networks [25–28]. Such models can also reproduce the expected course of the stochastic SIR model with constant infection and recovery rates on large configuration model networks with with several recent asymptotic proofs of convergence published [29–32]. Much work has also focussed on extending such methods to weighted [33] and dynamic [34] networks, as well as to models with arbitrary duration of the infectious period [35–37], with the common denominator that on clustered networks results from all approaches are only approximate.

Despite all these successes concerning SIR models (or related models such as SEIR, which includes an exposed period [17, 24]), the same is not generally true for infections without long-lasting immunity, with realistic demographic turnover or with significant viral mutation [38, 39]—i.e., the majority of pathogens of interest. Here we focus on STIs since the motivation for use of a network is strong [40]. These diseases are of major public health importance and the appropriate modelling framework (the SIS model on a network, also called the ‘contact process’ and frequently considered in theoretical studies) is the most challenging for approximation models to capture. To fully predict the dynamics and hence the impact of control on a range of sexually transmitted infections requires mathematical models that can account for both network structure of sexual partnership and the complications that arise from reinfection that is associated with SIS-type behaviour [40]. Here we consider three distinct approaches to capture the dynamic build-up of correlations between nearby individuals on the network—each approximation methodology has an associated integer that can be increased to achieve greater levels of accuracy. We stress, however, that our approach does not rely on special features of the SIS model but can be applied to the full spectrum of disease-dynamics models used to inform applied epidemiology and public health (including those with short-term immunity and hence SIRS-type dynamics).

Although the long-term aim is to utilise such approximation techniques to gain a clear understanding of the dynamics of STIs (as well as other infections that confer short-duration immunity) on realistic networks, we focus this paper on understanding when simple modelling techniques fail. One occasion when simple approaches fail is in the case of extreme heterogeneity [41–44]. Although risk-structure (or heterogeneity in network structure) is a highly important aspect of modelling STI—especially in terms of defining individual risk—we argue that many studies have focussed on this aspect [45, 46] and that it is usually possible to capture epidemiological effects of population heterogeneity by modestly increasing the system’s dimension through the introduction of multiple risk-groups (e.g. low- and high-risk behaviour).

**Fig 1:**
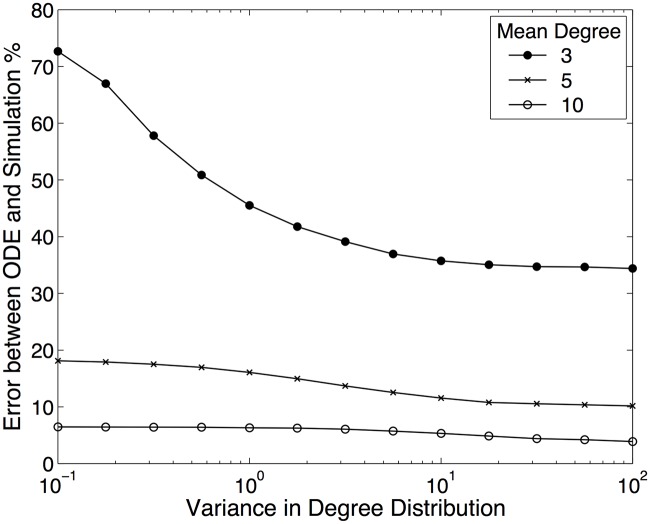
Comparison between risk-structured mean-field results and stochastic network simulations for SIS infection dynamics. The error is the relative percentage error between the risk-structured mean-field model (Supporting Information) and network simulation results for the mean prevalence of infection: $\text{Error}=100\times \|{\bar{I}}_{ODE}-{\bar{I}}_{network}\|/{\bar{I}}_{network}$. The degree distribution obeys *P*(*k*) = *ρ* exp(−*α*(*k* − *K*)^2^) for all *k* ≥ 2, where *α* and *K* are determined to give a desired mean and variance, and *ρ* is a normalising constant. Other methods of generating degree distributions give similar results. We insist on *k* ≥ 2 (for all nodes) as this generally ensures that the majority of network form a single giant component. The network is formulated using the Molly-Reed algorithm [47] with 100,000 nodes, but ensuring that there were no self-contacts and no multiple connections between individuals. We assume the recovery rate of individuals γ is scaled to be one, while the transmission rate across a contact scales with the mean number of contacts, $\tau =2/\bar{k}$. As such, the mean prevalence in both simulation and ODE models lies between 30 and 50%.

In contrast to the heterogeneous case, the impact of a limited number of contacts and the build-up of dynamical correlations in the state of neighbouring nodes is a less studied issue that presents deep conceptual challenges, especially for SIS dynamics. We therefore focus on developing a better understanding of this problem by ignoring many realistic features of STIs (we briefly comment on them in the discussion) and considering the idealised case of a homogeneous degree or ‘*k*-regular’ networks with low connectivity and hence greater importance of the link with each contact (in particular *k* = 3 and *k* = 2). In these remarkably simple networks, the effects of local correlations are at their strongest and are not masked by the impact of degree heterogeneity. To illustrate this concept we compare simple risk-structured mean-field (random-mixing) models (which account for degree heterogeneity within the network but not correlations that develop due to contact structure) with results from stochastic network simulations of SIS infection dynamics (Fig 1). This example demonstrates that when either the mean degree or the variance in the degree distribution increases, so the standard risk-structured ODE model provides a better fit to the simulated dynamics. The agreement between these simple models and simulations is worst for a homogeneous degree 3 network and hence it is this test scenario we predominantly consider throughout this paper.

## Methods

### Discussion of models

In this work, simulation models and traditional mean-field approximation models (that ignore network structure) represent two extremes in terms of analytic tractability and computational efficiency. Two approximate models for SIS-dynamics (Materials and Methods) have been developed that lie between these extremes: *pairwise* and *neighbourhood* approximations. Pairwise approximations [45, 48–50] consider the dynamic states of pairs of individuals that are connected in the network and hence capture some of the build-up of local correlations within the network. Neighbourhood approximations [22] have appeared more recently and can be conceptualised as a more sophisticated, though higher-dimensional, extension to the pairwise approximation. Neighbourhood approximations model the number of connected individuals of each type around a central individual; for SIS dynamics this is simply the number of *S* and *I* connected to a central individual of a given state. This means that neighbourhood models capture higher-order correlations within the network, as they effectively model multiple chains of three connected individuals sharing the same central node.

We consider methods to extend the pairwise and neighbourhood approaches; either increasing the size of the subgraph considered (e.g. going from modelling pairs to modelling triple motifs) or increasing the number of node states by counting infection events.

**Fig 2:**
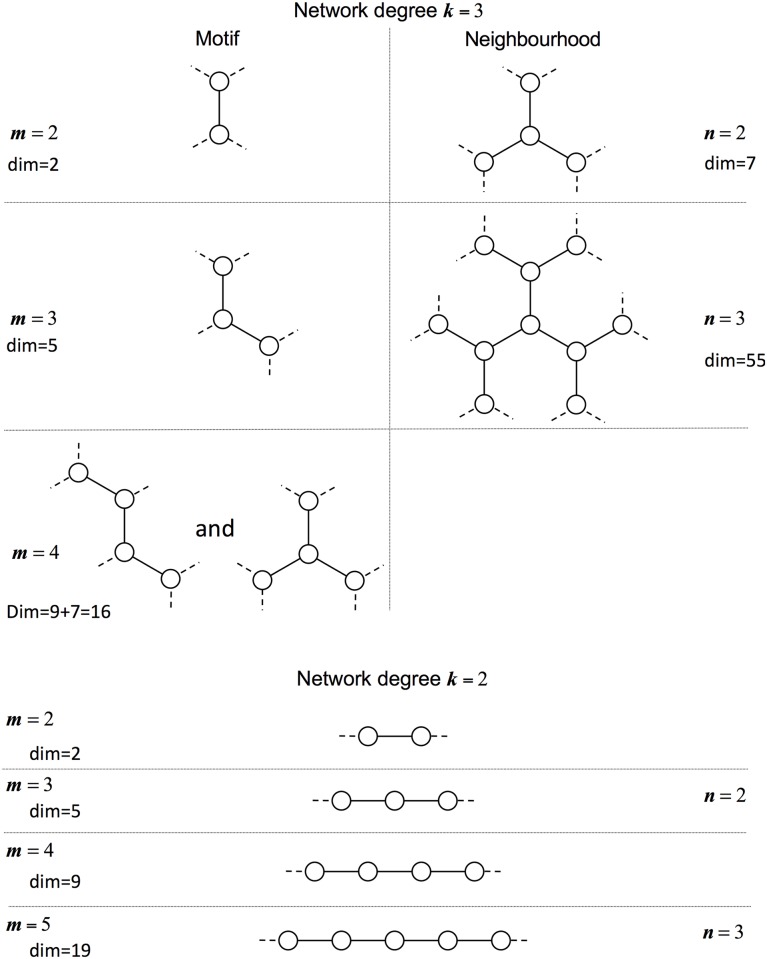
Subgraphs modelled by each approximation scheme. *k* denotes the (uniform) node degree and *m* or *n* indexing the size of the subgraph expansions for motif and neighbourhood schemes, respectively. Nodes within the subgraphs are shown and joined with solid lines; connections to other nodes (and hence where approximations are needed) are shown with dashed lines. The dimension of the associated ODEs for approximating the *SIS* dynamics is also given, accounting for symmetry and conservation. For degree *k* = 2 the dimension of the system can be calculated for a general *m* by considering the number of possible states with symmetries dim = 2^*m*−1^ + 2^*M*−1^ − 1 where *M* = ⌊(*m* + 1)/2⌋. Note, in this case,the natural relationship between the motif and neighbourhood models when *m* = 2*n* − 1.

Subgraph or motif expansions to the pairwise models track the dynamics of the possible states of increasingly larger motifs or subgraphs of *m* connected individuals within the network. Clearly as *m* becomes large, we precisely account for the full dynamics on larger sections of the network and hence expect our approximations to become more accurate. However with increasing *m* comes increasing number of motifs and also higher dimension dynamics. For degree *k* = 3 networks we consider motifs of size *m* = 1 (the standard mean-field model), *m* = 2 (the traditional pairwise model) as well as *m* = 3 and *m* = 4; for the special case of degree *k* = 2 networks, we are able to consider larger motifs up to *m* = 16 due to the linear structure of all *k* = 2 motifs (see Fig 2).

Similarly, it is feasible to expand the neighbourhood model, which we index by parameter *n*. Again we consider *n* = 1 to be the standard mean-field model, while *n* = 2 accounts for the states of all neighbours of a central individual, and expansions to neighbours of neighbours (*n* = 3) is also possible although of very high dimensional for networks of *k* = 3 or above.

The reinfection counting extension explicitly tracks the number of times an individual has been infected, effectively increasing the number of states for each individual (i.e. disease state × number of times infected). To create a finite system, we track the infection times up to a maximum of *L* (which now incorporates all those individuals infected *L* times or more). The motivation for this extension derives from a failure in traditional SIS pairwise models to account for the correlation between infected and newly recovered individuals. This extension should therefore improve the performance of the approximation model during the early stages of invasion when infection is rare. However, the long-term equilibrium dynamics when all individuals have been infected *L* times or more (assuming the infection persists), will be identical to that of the pairwise model.

**Fig 3:**
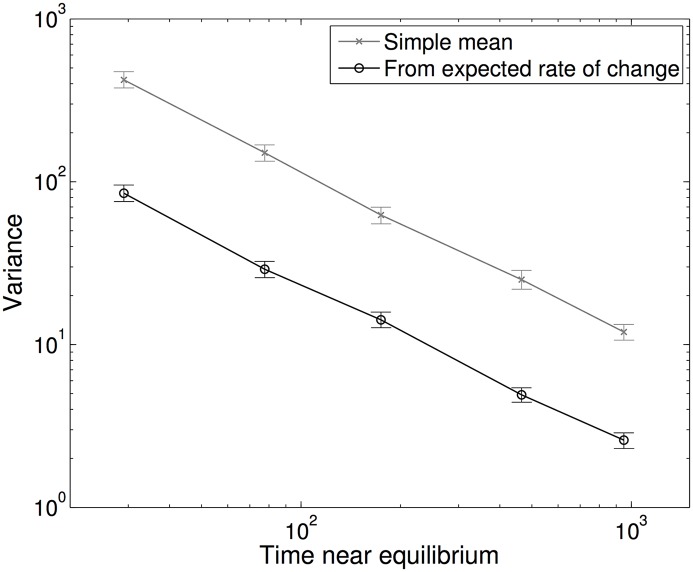
Variance about the true expected endemic level of prevalence. As calculated from very large simulations for two methods of determining the endemic prevalence for an SIS model on a network. Grey cross refer to taking the simple mean of the prevalence from time-series data, black circles are generated by fitting to the expected rates of change. Both are calculated once the simulation is close to its endemic state. Simulations are performed on a small network of 10,000 nodes for a relatively short time to highlight the differences; *k* = 3, *τ* = 1, γ = 1.

Although we take the simulation model as our gold-standard, deriving precise values for particular quantities is often computationally intensive and naive methods can be improved. For early epidemic growth rates, we generate a finite Cayley tree (thereby eliminating all clustering) and study the dynamics until infection hits an outer leaf. For endemic prevalence it is not possible to use a Cayley tree; any finite Cayley tree must have lower degree at the outer leaves which would influence the dynamics. Instead, we generate large networks using the Molloy-Reed (or configuration) algorithm [47], and ensure that self connections, multiple connections between nodes and short loops (of five or less connections) are removed by randomly shuffling connections. Moreover, far greater accuracy can be achieved when estimating quantities from simulation by realising that the expected rate of change of infection is determined by the state of the network and is given exactly by mechanistic models (such as Eq 3) where the variables are taken directly from the simulation. This allows us to remove some of the effects of stochasticity from the calculation. We therefore use this expected rate of change to directly calculate early growth rates, and use the long-term relationship between prevalence and expected rate of change to find the endemic equilibrium prevalence (see S1 Text). Fig 3 shows the advantage of this method, reducing the variance in our estimate of mean endemic prevalence and hence improving the accuracy of any fixed duration simulation.

### Mathematical definition of models

**Fig 4:**
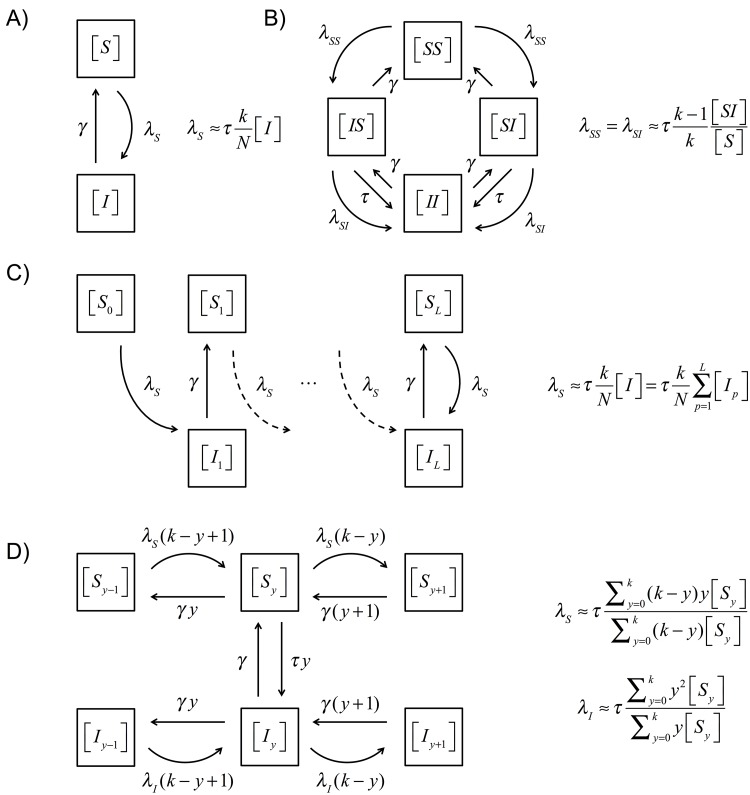
Models’ states and transition rates for the systems obtained from the: A) Mean-field approximation (Eq 3) B) Standard pairwise approximation (Eq 6); C) Mean-field approximation with reinfection counting (Eq 7) and D) Neighbourhood model with *n* = 2 (Eq 9). Curved arrows represent transitions due to a force of infection coming from outside the single node or pair. Dashed arrows represent flows to and from compartments the dynamics of which are tracked but that are not drawn explicitly.

We now layout in some detail the different approximation models used within this paper: mean-field; standard pairwise; reinfection counting; motif models; and neighbourhood models. The elements captured in each approximation are illustrated in Fig 4.

#### Full dynamics and notation

We consider individuals labelled with integers *i*, *j*, … ∈ {1, …, *N*} connected on a network with adjacency matrix ***A*** = (*A*_*i*,*j*_); where *N* is the number of nodes in the network, and *A*_*i*,*j*_ is one if nodes *i* and *j* are connected or zero otherwise. The state of individual *i* at time *t* is given by a Bernoulli random variable *X*_*i*_(*t*) taking the value *S*_*i*_ if *i* is susceptible and *I*_*i*_ if *i* is infectious. The events and rates of the full underlying dynamics are

$$\begin{array}{cc} S_{i} & \to I_{i}\;\text{at}\;\text{rate}\;\tau \sum_{j}A_{i,j}1_{\left\{X_{j}\left(t\right)=I_{j}\right\}}\;\text{,}\\ I_{i} & \to S_{i}\;\text{at}\;\text{rate}\;\gamma \;\text{,} \end{array}$$ (1)

where 1 is the indicator function. We use the pair-wise methodology and nomenclature developed by Keeling [23], where

$$\begin{array}{cc} {}[A] & :=E\left[\sum_{i}1_{\left\{X_{i}\left(t\right)=A_{i}\right\}}\right]\;\text{,}\\ \left[AB\right] & :=E\left[\sum_{i,j}1_{\left\{X_{i}\left(t\right)=A_{i}\&X_{j}\left(t\right)=B_{j}\&A_{i,j}=1\right\}}\right]\;\text{,} \end{array}$$ (2)

and similarly for larger structures.

#### Mean-field approximation

The following equations (and (Eq 5) below) can be shown to follow from (Eq 1) for any network [51]:

$$\frac{d[S]}{dt}=-\tau \left[SI\right]+\gamma \left[I\right]\;\text{,}\;\frac{d[I]}{dt}=\tau \left[SI\right]-\gamma \left[I\right]\;\text{.}$$ (3)

The mean-field approximation for a *k*-regular network takes these together with the assumption

[SI]≈(k/N)[S][I]. (4)

#### Standard pairwise approximation; *m* = 2

Here we take (Eq 3) together with

$$\begin{array}{cc} \frac{d[SS]}{dt} & =2\gamma [SI]-2\tau [SSI]\;\text{,}\\ \frac{d[SI]}{dt} & =\gamma [II]+\tau [SSI]-\tau [SI]-\gamma [SI]-\tau [ISI]\;\text{,} \end{array}$$ (5)

noting that [*IS*] = [*SI*] and [*II*] = *kN* − [*SS*] − 2[*SI*]. The standard pairwise approximation, typically attributed to Kirkwood [52], is

$$\left[ABC\right]\approx \frac{k-1}{k}\frac{\left[AB][BC\right]}{\left[B\right]}\;\text{.}$$ (6)

Following the work of [25, 30, 31] and [53], the approximation in (Eq 6) is exact for tree-like SIR epidemics when *B* = *S*, which is sufficient to guarantee the exactness of (Eq 5).

#### Systematic approximation 1: Reinfection counting

Here we count the number of times *p* that an individual has been infected up to a maximum of *L*. Using a straightforward notation (defined fully in S1 Text) the dynamics (Eq 3) become

$$\begin{array}{cc} \frac{d[S_{p}]}{dt} & =-\tau \left[S_{p}I\right]+\gamma \left[I_{p}\right]\;\text{,}\;\forall p\text{,}\\ \frac{d[I_{p}]}{dt} & =\left\{\begin{array}{cc} \tau \left[S_{p-1}I\right]-\gamma \left[I_{p}\right]\;\text{,} & p<L\;\text{,}\\ \tau \left[S_{p-1}I\right]+\tau \left[S_{p}I\right]-\gamma \left[I_{p}\right]\;\text{,} & p=L\;\text{,} \end{array}\right. \end{array}$$ (7)

where the subscript refers to the number of times an individual has been infected. Similarly, the dynamics (Eq 5) become

$$\begin{array}{cc} \frac{d[S_{p}S_{q}]}{dt} & =\gamma \left(\left[S_{p}I_{q}\right]+\left[I_{p}S_{q}\right]\right)-\tau \left(\left[S_{p}S_{q}I\right]+\left[IS_{p}S_{q}\right]\right)\;\text{,}\;\forall p,q\;\text{,}\\ \frac{d[S_{p}I_{q}]}{dt} & =\gamma \left[I_{p}I_{q}\right]+\tau \left[S_{p}S_{q-1}I\right]-\tau \left[S_{p}I_{q}\right]\\ & \;-\gamma \left[S_{p}I_{q}\right]-\tau \left[IS_{p}I_{q}\right]\;\text{,}\;q<L,\forall p\;\text{,}\\ \frac{d[S_{p}I_{L}]}{dt} & =\gamma \left[I_{p}I_{L}\right]+\tau \left[S_{p}S_{L-1}I\right]+\tau \left[S_{p}S_{L}I\right]-\tau \left[S_{p}I_{L}\right]\\ & \;-\gamma \left[S_{p}I_{L}\right]-\tau \left[IS_{p}I_{L}\right]\;\text{,}\;\forall p\;\text{.} \end{array}$$ (8)

We close these using the triple approximation (Eq 6), with the added notation that lack of a subscript refers to a sum over all possible infection counts (e.g. [*S*_*p*_ *I*] = ∑_*q*_[*S*_*p*_ *I*_*q*_] or [*S*_*p*_ *S*_*q*_ *I*] = ∑_*r*_[*S*_*p*_ *S*_*q*_ *I*_*r*_]).

#### Systematic approximation 2: Motif models

To carry out a motif-based expansion at order *m* we write down the dynamics for the complete subgraphs of size *m*, whose rates of change will be functions of the complete subgraphs of sizes *m* and *m* + 1; these *m* + 1 subgraphs are then approximated using the general form of the Kirkwood closure, where the size-*m* motifs in the size-(*m* + 1) motif are multiplied, and divided through by the over-counted size-(*m* − 1) motifs, then divided through by the over-counted *m* − 2 motifs and so on until the size-1 motifs are reached. This involves a large amount of notational development that is given in full for *m* = 3 in [54], and for the special case of *k* = 2 in S1 Text.

#### Systematic approximation 3: Neighbourhood model, *n* = 2

Suppose we write [*A*_*y*_] for the expected number of nodes in state *A* with *y* infectious neighbours, then the dynamics that follow from (Eq 1) are

$$\begin{array}{cc} \frac{d[S_{y}]}{dt} & =\gamma \left[I_{y}\right]+\lambda_{S}\left(k+1-y\right)\left[S_{y-1}\right]+\gamma \left(y+1\right)\left[S_{y+1}\right]\\ & \;-\tau y\left[S_{y}\right]-\lambda_{S}\left(k-y\right)\left[S_{y}\right]-\gamma y\left[S_{y}\right]\;\text{,}\\ \frac{d[I_{y}]}{dt} & =\tau y\left[S_{y}\right]+\lambda_{I}\left(k+1-y\right)\left[I_{y-1}\right]+\gamma \left(y+1\right)\left[I_{y+1}\right]\\ & \;-\gamma \left[I_{y}\right]-\lambda_{I}\left(k-y\right)\left[I_{y}\right]-\gamma y\left[I_{y}\right]\;\text{.} \end{array}$$ (9)

Where the forces of infection (λ_*S*_ and λ_*I*_) refer to the rate of infection acting on a (susceptible) neighbour of the central node (in state S or I respectively). We then approximate the forces of infection by making the susceptible neighbour the centre of a new neighbourhood and looking for the distribution of consistent neighbourhoods:

$$\lambda_{S}\approx \tau \frac{\sum_{y=0}^{k}\left(k-y\right)y\left[S_{y}\right]}{\sum_{y=0}^{k}\left(k-y\right)\left[S_{y}\right]}\;\text{,}\;\lambda_{I}\approx \tau \frac{\sum_{y=0}^{k}y^{2}\left[S_{y}\right]}{\sum_{y=0}^{k}y\left[S_{y}\right]}$$ (10)

This approach can be extended to order *n* > 2 by considering the dynamics in two parts: firstly the dynamics internal to each extended neighbourhood; secondly the force of infection on any susceptible individual at the edge of the extended neighbourhood. Again these forces of infection are found by considering consistent overlaps of the neighbourhoods centred on the susceptible neighbour under consideration.

## Results

**Fig 5:**
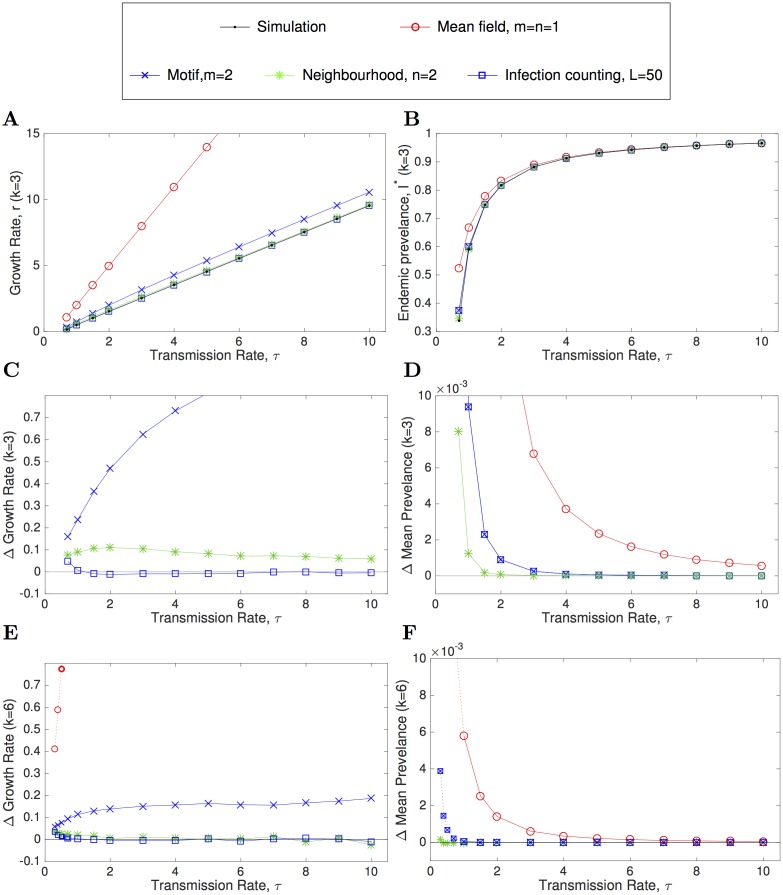
Growth rate (left column) and prevalence (right column) from a range of approximation models. Standard SIS model (Eq 3), *m* = *n* = *L* = 1); pairwise (motif) model (Eq 5, *m* = 2); neighbourhood model (Eq 9, *n* = 2); and reinfection counting (Eqs 7 and 8, *L* = 50). These are compared to findings from direct numerical simulation (Eq 1, black dots) for an SIS infection on a simple *k*-regular network. The upper panels show the absolute value of (A) the growth rate and (B) the prevalence as the transmission rate across a link (*τ*) is varied, for *k* = 3. The lower four panels show the difference between the approximations and the results from stochastic simulation (C and D: *k* = 3; E and F: *k* = 6). For *k* = 6 (panels E and F) additional smaller *τ* values (joined with dotted lines) are considered as the critical value that allows persistence is reduced compared to *k* = 3. Numerical simulations are performed on a 100,000 node network and run for sufficiently long that confidence intervals are negligible. A recovery rate γ = 1 is assumed throughout.

We begin by comparing the growth rates from four approximation models (mean-field, pairwise (motif *m* = 2), pairwise with reinfection counting (*L* = 50) and neighbourhood (*n* = 2), see S1 Text) with those from stochastic simulations on a Cayley tree, for different values of the transmission rate *τ* substantially above the critical value that permits successful invasion (Fig 5A, 5C and 5E). Unsurprisingly, the standard ODE model that ignores all elements of network structure (and hence ignores the negative *S*-*I* correlations that build-up and reduce transmission within a network) vastly over-estimates the early growth rate. Including some element of local structure, such as that captured by the pairwise (motif *m* = 2) model substantially improves the prediction of the growth rate but still overestimates compared to the simulated value. Finally, adding additional structure, either in terms of the reinfection counting or neighbourhood expansion enhances the accuracy. On closer inspection (Fig 5C and 5E) we observe that away from the critical invasion point, the reinfection counting model provides a highly accurate prediction of the early growth rate, outperforming all other approximation methods. In addition, as indicated by Fig 1, we find that for higher degree networks (*k* = 6, Fig 5E) all models, even the standard mean-field ODE model, provide a more accurate estimate of the true behaviour.

Turning our attention to the prevalence of infection (Fig 5B, 5D and 5F), it is clear that all approximation models (even the standard mean-field model) perform reasonably well when comparing their equilibrium values with the numerical estimates of the expected prevalence. As mentioned before, we also note that the standard pairwise model (*m* = 2) and reinfection counting pairwise model (for any *L*) have the same equilibrium prevalence—in the reinfection counting model all individuals will eventually be infected more than *L* times, thereby reaching the upper limit. However, even taking *L* very large, the same quasi-equilibrium prevalence is reached even before a significant fraction of individuals hit the upper reinfection counting limit *L*. This is related to the loss of local correlation structure as the network becomes saturated with infection and paths of infection meet through medium and long loops within the network.

Comparing more closely results from the approximation models against simulated prevalence (Fig 5D and 5F) shows that the neighbourhood model (*n* = 2) outperforms the pairwise models (*m* = 2). This is to be expected as the neighbourhood model captures higher-order spatial structure within the network, effectively capturing the status of *k* + 1 connected individuals. However, all approximation models perform worse as the expected prevalence drops and the critical transmission rate is approached.

**Fig 6:**
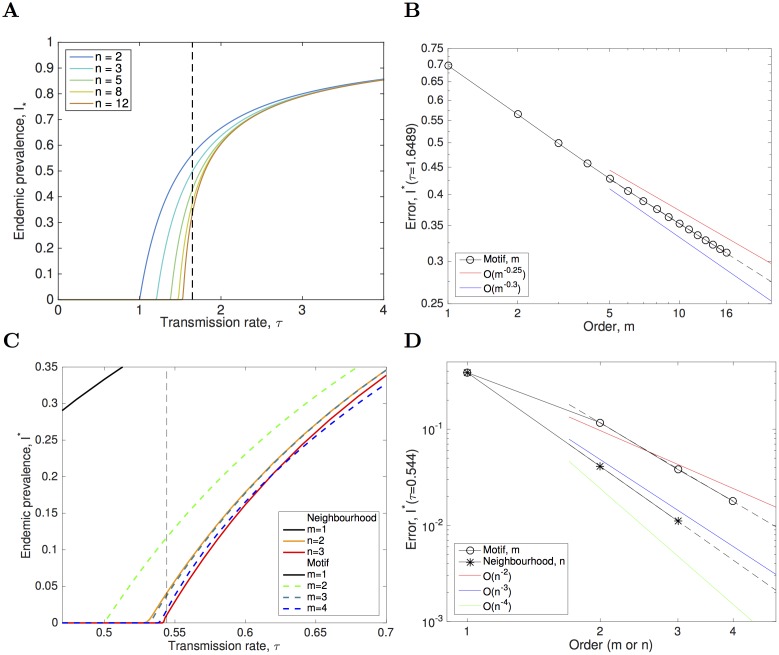
Endemic prevalence of infection for an SIS model for successively higher-order approximations. The focus is on the the value of the transmission rate near the critical point (shown as vertical dashed line), when *k* = 2 (A and B) and *k* = 3 (C, D). The errors at the critical point for the *k* = 2 degree network (B) show very slow convergence in the order of the approximation *m*; neighbourhood and motif model results coincide when *m* = 2*n* − 1 and are not reported. For *k* = 3 degree network, the errors at the estimated critical point (D) show rapid convergence as the models are extended to higher order (increasing *m* and *n*). The error in the motif model scales like *O*(*m*^−2.71^) while for the much higher dimensional neighbourhood model the error scale like *O*(*n*^−3.24^).

This comparison raises the question of how the motif and neighbourhood approximations perform as *m* and *n* are increased, incorporating more of the local network. We consider two cases. Firstly, *k* = 3 where only limited extensions of the models are feasible (*m* = 3, *m* = 4 and *n* = 3) as the dimension of the systems rapidly becomes large and the mathematical formulations are unwieldy. Secondly *k* = 2 (which we note is a special case [55, 56]) where neighbourhood and motif models are equivalent for *m* = 2*n* − 1, and where we can readily extend the approximation methods to extremely high orders (S1 Text). Fig 6 demonstrates the impact of taking these higher order approximations. Considering the *k* = 2 case (when the network is a linear system), increasing the order (*m* = 1 to *m* = 16) leads to a drop in endemic prevalence and convergence of the critical transmission rate to the estimated value (vertical line). At the critical transmission value (estimated as *τ*_*C*_ ≈ 1.6489 [57]) the error scales extremely slowly with the order of the model (approximately *O*(*m*^−0.271^)), Fig 6B). When returning to the case *k* = 3 that has been the main focus of this work (where we estimate *τ*_*C*_ = 0.544 from numerous large scale simulations) both the approximation methods behave far better (motif error ∼*O*(*m*^−2.7^); neighbourhood error ∼*O*(*n*^−3.2^)), and offer reliable predictions of endemic prevalence even quite close to the critical point as the order of the approximations increases.

## Discussion

Moment closure approximations for the spread of infections on networks can be highly informative, especially when uncertainty in the underlying network structure precludes detailed simulation of a specific case. By generating relatively simple, tractable models (in the form of ODEs), an intuitive understanding can be developed for the spread of infection that does not rely on precise measurement of network structure. This approach has been highly successful for infections with SIR-type dynamics [23–25], where recovery leads to lifelong protection; however, for infections that obey the SIS paradigm and can therefore be contracted multiple times this closure approach does not have the same level of precision [40, 45, 48, 49]. Most sexually transmitted infections are well approximated by SIS-type dynamics, and modelling sexually transmitted infections requires an appreciation of the dynamic implications of the sexual contact network, due to the relatively low numbers of sexual contacts at any time. Therefore, although closure approximations for SIS-type infections on networks is highly challenging, it is nevertheless an area of considerable applied importance. Several other recent studies have considered the behaviour of SIS models on networks [42, 45, 49–51, 54, 58–61] showing that this is a field of active research where there are substantial challenges in establishing rigorous analytical results and in matching approximations, simulations and real data.

In this paper we have mainly focussed on homogeneous random networks where each individual has exactly *k* = 3 contacts, and all contacts are considered bi-directional. This restricts our attention to the highly challenging case of small and homogeneous degree, as higher mean degree or greater heterogeneity leads to infection prevalences that are closer to mean-field predictions that ignore the local correlations that arise from network structure. For homogeneous random networks of this type we show that, as expected, pairwise approximations (that consider the state of two connected individuals) outperform standard models (that ignore any correlations within the network), while neighbourhood-based models (originally called ‘effective degree models’ [22]—that consider the state of all neighbours around a central individual) outperform pairwise models (Fig 5A and 5B), and in turn extended neighbourhood models (that consider neighbours of neighbourhoods) are even more accurate (Fig 6C). This is unsurprising since closing the approximation at higher orders, and therefore essentially modelling more of the underlying local behaviour, is always likely to provide a more accurate description of the population-scale dynamics.

We also investigated extensions to the standard closure models, including a count of the number of times an individual has been infected. This removes some of the inaccuracies that pairwise (and other) approximation models suffer from when trying to capture the early stages of infection in a largely susceptible population. The results of this improvement to the pairwise model generates far better predictions for the early growth-rate of infection, offering a substantial improvement over both standard pairwise models and neighbourhood models (Fig 5). It would therefore seem prudent, although dimensionally-challenging, to combine reinfection counting with closure models that operate at the whole neighbourhood scale (or even larger) thereby enabling an approximation to both the early and endemic dynamics. However, it may be far simpler to use the model most appropriate to the setting, depending on whether it is early growth or endemic prevalence that is required.

With moment closure models there is always the temptation to include higher order terms in the approximation and close at one order higher. We considered higher-order approximations to SIS dynamics on both *k* = 3 and *k* = 2 networks, noting that the SIS model on *k* = 2 is identical to classic 1-dimensional contact process [55, 56]. For the 1-dimensional *k* = 2 case, we are able to extend the modelling approach to much higher orders, but find that these closures still overestimate the prevalence near to the critical point. For the *k* = 3 network, we are only able to extend the neighbourhood model one additional step (considering neighbours of neighbours). However, as we capture the status of more contacts around the central individual, this extended neighbourhood model provides a very accurate approximation to the SIS behaviour, although it is complex to construct and relatively high-dimensional.

Throughout we have focused on expanding our approximations to ever higher orders, but the upper bounds to what can be achieved differ between methods. For the reinfection counting model (where dimension of the system is 2*L*^2^ − 1 and hence grows relatively slowly with *L*) taking *L* past 50 had very little effect, so further expansion was irrelevant. For degree *k* = 2 networks, where motif and neighbourhood expansions are equivalent, the limiting factor was the dimension of the system. The ODEs were simulated up to *m* = 16 at which point the dimension is 2^*m*−1^ + 2^*M*−1^ − 1 = 32,895 (where *M* = ⌈(*m* + 1)/2⌉ = 8). For degree *k* = 3 the main limitation is not the dimension of the system but the complexity of closure approximations which utilise the probabilities of overlapping subgraphs; considering approximation higher than *m* = 4 or *n* = 3 is possible, but the gains in accuracy may not be worth the considerable effort.

Finally, we consider the fact that sexual networks are highly heterogeneous (with some individuals having many more life-time partners than others [3, 62]). This risk heterogeneity is important for understanding who becomes infected, but the action of this heterogeneity is readily captured by traditional risk-structured mean-field models [63, 64] that ignore network correlations (Fig 1). Extending all the models discussed in this paper to capture degree heterogeneity is possible although one needs to specify, in addition to the degree distribution, a degree correlation matrix—the choice of which can have a dramatic impact on the disease dynamics [9]. Furthermore, the dimensionality of the system can rapidly exceed currently available computational resources due to the combinatorial number of possible configurations, especially for the neighbourhood model. For example, considering neighbourhood models *n* = 2: 5 equations are needed for *k* = 2 networks and 7 equations for *k* = 3 networks; however when the degree is heterogeneous far more equations are required, in a network where all nodes are degree 1 or 2 then 27 equations are needed but if nodes can be degree 1, 2 or 3 then the number of equations rises to 165. For neighbourhood models approximated at the next order (*n* = 3) the effect is even more dramatic; a heterogeneous networks where nodes can be degree 1, 2 or 3 requires 65,015 equations. We note, however, realistic sexual networks may have a large proportion of population with relatively few connections and where infections spread poorly; this leads us to believe that large sections of sexual networks may behave more like the low degree (*k* = 2 or *k* = 3) situations considered here where there is a strong need to accurately capture network correlations. As heterogeneity increases, pairwise models, which do not suffer such a pathological growth in dimensionality, become relatively accurate and, as was illustrated in Fig 1, even simple ODE models can be highly effective at capturing the aggregate prevalence in highly heterogeneous populations. Depending on the applied problem, we argue that a combination of the systematic approximations here can be used as a trade-off between accuracy and computational complexity.

Even if degree heterogeneity is captured by models, the network structure and infection dynamics used here are extreme simplifications of real-world behaviour: in particular, sexual networks are dynamic (with most individuals practising serial monogamy [45]), and the natural history of infection is often far from the Markovian process with only two states (S and I) discussed here. In reality, for most STIs, there is likely to be a latent period following infection; detection, treatment and recovery will follow (non-Markovian) processes; and treatment is likely to offer some limited protection. These facets will act to prevent rapid reinfection of individuals, which, like heterogeneity, should improve the accuracy of approximation models.

Future work should clearly focus on developing more sophisticated and realistic network-based simulation models for STIs and comparing these to a range of approximation methods; however, we believe that the careful exploration of the accuracy of approximate models performed here is a key step in this process.

## Supporting Information

S1 Text Technical model definitions and methods. This Supplementary Material contains further technical model definitions and methods. Equations are numbered in continuation from the main text. Figures references and citations refer to the main text.
(PDF)
