# Supplementary material for: Systematic Approximations to Susceptible-Infectious-Susceptible Dynamics on Networks
Source: PLoS Comput Biol. 2016 Dec 20;12(12):e1005296. doi: 10.1371/journal.pcbi.1005296 (PMC5283726; doi:10.1371/journal.pcbi.1005296)
Supplement: S1 Text — This Supplementary Material contains further technical model definitions and methods. Equations are numbered in continuation from the main text. Figures references and citations refer to the main text. (PDF) [file pcbi.1005296.s001.pdf]

Systematic Approximations to Susceptible-Infectious-Susceptible Dynamics on Networks

M. J. Keeling[1,2,3*], T. House[4,1,2], A. J. Cooper[5], L. Pellis[1,2],

Technical details: model definitions and methods

1 Zeeman Institute: SBIDER, University of Warwick, Coventry, CV4 7AL, United Kingdom.
2 Mathematics Institute, University of Warwick, Coventry, CV4 7AL, United Kingdom.
3 School of Life Sciences, University of Warwick, Coventry, CV4 7AL, United Kingdom.
4 School of Mathematics, University of Manchester, Manchester, M13 9PL, United Kingdom.
5 School of Engineering, University of Warwick, Coventry, CV4 7AL, United Kingdom.

* M.J.Keeling@warwick.ac.uk

Risk-structured mean-field models

For risk-structured mean-field models (as used in Figure 1 of the main paper), the standard mean-field equations (Eq 3, with approximation 4) can be modified to capture the status of individuals with a given degree [60, 61]:

$$\frac{\mathrm{d}[I_k]}{\mathrm{d}t} = -\frac{\mathrm{d}[S_k]}{\mathrm{d}t} = \tau k[S_k]\sum_j \sigma_{k,j}[I_j] - \gamma[I_k] \ . \tag{11}$$

where $[S_k]$ and $[I_k]$ refer to the number of susceptible and infected nodes of degree k, and $\sigma_{k,j}$ is the probability that a randomly chosen contact of a degree-k node has degree j. In the Molloy-Reed- (or configuration-)type networks [48], this degree-degree structure is independent of k and is given by:

$$\sigma_{k,j} = \frac{jN_j}{\sum_i iN_i}$$

where $N_j = [S_j] + [I_j]$ is the number of nodes of degree j.

Alternative pairwise formulation

Instead of formulating the pairwise equations by considering the dynamics imparted by the larger triples, an alternative is to consider the force of infection λ acting on a susceptible member of the pair from outside the pair.

$$\begin{aligned}
\frac{\mathrm{d}[SI]}{\mathrm{d}t} &= \gamma[II] + \lambda[SS] - \tau[SI] - \gamma[SI] - \lambda[SI], \\
\frac{\mathrm{d}[SS]}{\mathrm{d}t} &= 2\gamma[SI] - 2\lambda[SS], \\
\frac{\mathrm{d}[II]}{\mathrm{d}t} &= 2\tau[SI] + 2\lambda[SI] - 2\gamma[II].
\end{aligned} \tag{12}$$

If we then assume that this external force of infect comes from any of the remaining $k-1$ connections not accounted for in the pair we have:

$$\lambda \approx \tau \frac{k-1}{k} \frac{[SI]}{[S]},$$

and we regain the formulation of (Eq 5) with Kirkwood's closure (Eq 6); this methodology is extended below to consider larger motifs when $k = 2$.

Higher-order approximations

2-regular network: motif and neighbourhood models

For the case where $k = 2$ [56, 57], due to the linear arrangement of the nodes in the network, it is relatively simple to derive higher-order approximations that explicitly account for larger sections of the network. Keeping with the pairwise notation, and considering n connected nodes we have:

$$\frac{\mathrm{d}[\mathbf{X} = (X_1 X_2 \ldots X_n)]}{\mathrm{d}t} = \sum_{\mathbf{Y}} \boldsymbol{Q}_{\mathbf{X},\mathbf{Y}}[\mathbf{Y}] + \lambda^1_{\mathbf{X}}[S X_2 \ldots X_n] + \lambda^n_{\mathbf{X}}[X_1 \ldots X_{n-1} S] \tag{13}$$

where $X_1, \ldots, X_n \in \{S, I\}$. The matrix $\boldsymbol{Q}$ captures the dynamics that are internal to the n-states considered in the motif; that is, rates of recovery and transmission due to the arrangement of the n node states. Again the impact of nodes external to these states is formulated as an external force of infection ($\lambda^1_{\mathbf{X}}$ and $\lambda^n_{\mathbf{X}}$ referring to the external force of infection acting on either end of the set of n nodes) by considering an overlapping configuration, identical to that of the n-node motif, but shifted by one position to capture the neighbours of each node at its edge:

$$\lambda^1_{\mathbf{X}} \approx \begin{cases} -\tau \dfrac{[ISX_2 \ldots X_{n-1}]}{[SSX_2 \ldots X_{n-1}] + [ISX_2 \ldots X_{n-1}]} & \text{if } X_1 = S \\ \tau \dfrac{[ISX_2 \ldots X_{n-1}]}{[SSX_2 \ldots X_{n-1}] + [ISX_2 \ldots X_{n-1}]} & \text{if } X_1 = I \\ 0 & \text{otherwise.} \end{cases}$$

$$\lambda^n_{\mathbf{X}} \approx \begin{cases} -\tau \dfrac{[X_2 \ldots X_{n-1}SI]}{[X_2 \ldots X_{n-1}SS] + [X_2 \ldots X_{n-1}SI]} & \text{if } X_n = S \\ \tau \dfrac{[X_2 \ldots X_{n-1}SI]}{[X_2 \ldots X_{n-1}SS] + [X_2 \ldots X_{n-1}SI]} & \text{if } X_n = I \\ 0 & \text{otherwise.} \end{cases}$$

The number of ODEs in this formulation grows exponentially with n (the number of ODEs is 2^n ignoring symmetries), and when n is large there are frequent numerical difficulties with calculating the external force of infection (λ^1 and λ^2) due to small terms in the denominator. An alternative is to use an iterative solution where in each step the different forces of infection are fixed, and depend on the equilibrium density in the previous step. Thus for step s we have:

$$\frac{\mathrm{d}[\mathbf{X}]^s}{\mathrm{d}t} = \sum_{\mathbf{Y}} \boldsymbol{Q}_{\mathbf{X},\mathbf{Y}}[\mathbf{Y}]^s + (\lambda^1_{\mathbf{X}})^{s-1}[SX_2 \ldots X_n]^s + (\lambda^n_{\mathbf{X}})^{s-1}[X_1 \ldots X_{n-1}S]^s$$

This is now a linear problem and can therefore be solved with great precision and efficiency by calculating the dominant eigenvalue of the system; by starting with $\lambda^0 = \tau$ ensures that each approximation is an over-estimate and iterations converge on the true equilibrium of the system .

3-regular network: motif model

In a motif-based expansion, we expand the epidemic dynamics in motifs (connected subgraphs) of size-1 up to size-$(m+1)$. We then approximate the size-$(m+1)$ motifs in terms of the smaller motifs using Kirkwood's closure,

$$(m+1)\text{-motif count} \approx \cfrac{C_m \times n\text{-motifs in set of } (m+1)\text{-motifs}}{C_{m-1} \times \cfrac{\text{Overcounted } (m-1)\text{-motifs in set of } m\text{-motifs}}{\cfrac{\vdots}{C_1 \times \text{Overcounted nodes in set of pairs}}}} \tag{14}$$

although other closures are possible. For the 2-regular graph, the motif model for odd m is equivalent to a neighbourhood model, but at higher connectivity differences emerge. The unclosed equations contain very many terms, but can be written down in a straightforward notation as follows:

$$\begin{aligned}
\frac{\mathrm{d}}{\mathrm{d}t}[S] &= -\tau[S\text{–}I]\cdots \\
\frac{\mathrm{d}}{\mathrm{d}t}[S\text{–}S] &= -2\tau\,[S\text{–}S\text{–}I]\cdots \\
\frac{\mathrm{d}}{\mathrm{d}t}\,[S\text{–}S\text{–}S] &= -2\tau\,[S\text{–}S\text{–}S\text{–}I]\,-\tau\,[S\text{–}\overline{S\text{–}S\ \ I}]\cdots \\
\frac{\mathrm{d}}{\mathrm{d}t}\,[S\text{–}S\text{–}S\text{–}S] &= -2\tau\,[S\text{–}S\text{–}S\text{–}S\text{–}I]\,-2\tau\,[S\text{–}\overline{S\text{–}S\text{–}S\ \ I}]\cdots \\
\frac{\mathrm{d}}{\mathrm{d}t}\,[S\text{–}\overline{S\text{–}S\ \ S}] &= -3\tau\,[S\text{–}\overline{S\text{–}S\ \ S}\text{–}I]\cdots\ ,
\end{aligned} \tag{15}$$

where the lines above the node-states (S and I terms) refer to network connections that are present in these higher-order motifs. We note that for a three-regular graph ($k = 3$) only a limited number of motifs need to be considered. For four connected nodes only two motif configurations are possible without clustering: ⊓ and ⇖; while for five connected nodes again only two configurations need considering: ⊓⌞ and ⇖⟍. For higher degree regular graphs ($k > 3$) more complex motifs (such as the four-star ⟋⇖) are required although these can be approximated in the same manner. Applying the closure (Eq 14) to (Eq 15) at different stages gives the following set of closures, where motif structures in square brackets (e.g. [–], [∧] or [⊓⌞]) refer to the number of motifs of each type regardless of node status.

Mean field ($n = 1$):

$$[A\text{–}B] \approx \frac{[-]}{[\bullet]^2}[A][B]\ . \tag{16}$$

Pairwise ($n = 2$):

$$[A\text{–}B\text{–}C] \approx \frac{[\wedge][\bullet]}{[-]^2}\frac{[A\text{–}B][B\text{–}C]}{[B]}\ . \tag{17}$$

Triplewise ($n = 3$):

$$
\begin{aligned}
[A\text{–}B\text{–}C\text{–}D] &\approx \frac{[\sqcap][-]}{[\wedge]^2}\frac{[A\text{–}B\text{–}C]\,[B\text{–}C\text{–}D]}{[B\text{–}C]}\ , \\
[A\text{–}\overline{B\text{–}C\ \ D}] &\approx \frac{[\nwarrow][-]^3}{[\wedge]^3[\bullet]}\frac{[A\text{–}B\text{–}C]\,[C\text{–}B\text{–}D]\,[A\text{–}B\text{–}D]}{[B\text{–}C][A\text{–}B][D\text{–}B]} \times [B]\ .
\end{aligned}
\tag{18}
$$

Quad-wise ($n = 4$):

$$
\begin{aligned}
[A\text{–}B\text{–}C\text{–}D\text{–}E] &\approx \frac{[\sqcap\!\llcorner][\wedge]}{[\sqcap]^2}\frac{[A\text{–}B\text{–}C\text{–}D]\,[B\text{–}C\text{–}D\text{–}E]}{[B\text{–}C\text{–}D]}\ , \\
[A\text{–}\overline{B\text{–}C\text{–}D\ \ E}] &\approx \frac{[\nwarrow\!\diagdown][\wedge]^3[\bullet]^2}{[\sqcap]^2[\nwarrow][-]^2}\frac{[D\text{–}C\text{–}B\text{–}E]\,[D\text{–}C\text{–}B\text{–}A]\,[A\text{–}\overline{B\text{–}C\ \ E}]}{[B\text{–}C\text{–}D]\,[A\text{–}B\text{–}C]\,[C\text{–}B\text{–}E]} \times \frac{[B\text{–}C]^2}{[B][C]}\ .
\end{aligned}
\tag{19}
$$

The number of motifs in an unclustered k-regular graph with N nodes are the following:

$$
\begin{aligned}
&[\bullet] = N\ , && [-] = Nk\ , \\
&[\wedge] = Nk(k-1)\ , && [\nwarrow] = Nk(k-1)(k-2)\ , \\
&[\sqcap] = Nk(k-1)^2\ , && [\sqcap\!\llcorner] = Nk(k-1)^3\ , \\
&[\nwarrow\!\diagdown] = Nk(k-1)^2(k-2)\ .
\end{aligned}
\tag{20}
$$

3-regular network: neighbourhood model

When $k = 3$ it is only feasible to extend the neighbourhood approximation to next nearest neighbours ($n = 3$). This entails considering the central node, its three neighbours and then the state of the two additional nodes connected to each of these neighbours (Figure 2). Ignoring symmetries which could be used to reduce the dimensionality of the system, this extended neighbourhood model requires 432 ODEs. The calculation of the external force of infection is also more complex, both due to the number of overlapping nodes that need to be considered, but also because some of the nodes in the overlapping configuration do not play an active role in the infection being considered, but must still feature in the calculation. However, with careful bookkeeping the set of ODEs can be generated in a similar manner to those for the simpler neighbourhood model.

Extension to the degree-heterogeneous case

Considering the case where the network has heterogeneous degree distribution, the extension of the neighbourhood model is straightforward but requires us to index the state variables with the degree of the central node in the neighbourhood, e.g. write $[A^k_y]$ for the expected number of nodes of degree k in state A with y infectious neighbours and then (Eq 9) are unchanged. It is also straightforward to carry out reinfection counting for generalised models as another index for the state variables.

For motif-based expansion, however, the generalisation is not so obvious. One possibility is to label state variables with the degrees of the nodes in question as in [46] and for the neighbourhood model. Alternatively, one can consider a generalisation that does not involve extra indexing, replacing the pairwise moment closure (Eq 6) with

$$
[ABC] \approx \frac{[\wedge][\bullet]}{[-]^2}\frac{[AB][BC]}{[B]}\ , \tag{21}
$$

where now $[\bullet]$ is the expected number of nodes in the network, $[-]$ the expected number of pairs, $[\wedge]$ the expected number of 2-stars and so on.

Motivation for the reinfection counting pairwise model

One failing of the pairwise model (Eq 5) occurs during the early phase of disease invasion when the density of infected individuals is low. In this phase, and due to the localness of transmission, we would expect all pairs and triples containing one or more infected individuals to occur with a probability that is of the same order (i.e. $[IX] = O([I])$, $[IXY] = O([I])$, $[XIY] = O([I])$ and all symmetries of these quantities, for all $X, Y \in \{S, I\}$). This is because even triples such as $[III]$ can be created from a single infected individual by two relatively likely events – infection to either side before recovery. Hence all pairs and triples containing infection should occur at the same order – this highlights the strength of correlations in the early dynamics as without the action of such correlations triples such as $[III]$ would be vanishingly rare.

However this early scaling behaviour is not captured by the triple closure for one particular (but common) case. Examining the Kirkwood closure we have:

$$[ISI]_K \approx \xi \frac{[IS][SI]}{[S]} \approx \xi \frac{O([I])O([I])}{O(1)} = O([I]^2)$$

This failing is because the closure approximation is trying to put together two unlikely pairs ($[IS]$ and $[SI]$) without accounting for the fact that this triple can be formed from an $[III]$ triple where the central individual recovers back to the susceptible state. A similar failure will occur for the neighbourhood approximation when performing the closure that gives the force of infection (i.e. the force of infection on a susceptible contact of a central susceptible individual).

One way of ensuring the true scaling during this early phase is to differentiate between susceptible individuals that have never been infected and those that have recovered from infection; however this just delays the problems. We therefore choose to index individuals by the number of times they have been infected (i.e. $[S_p]$ and $[I_p]$ for individuals infected p times). Hence all individuals start life as S_0, then when first infected move into the I_1 class, then on recovery they move into the S_1 class, than on subsequent infection they progress into the I_2 class, etc. This produces an infinite cascade of states, which we truncate to make numerical progress. We fix an upper limit L, which determines the size of this cascade; as such, individuals of type S_L on infection produces individuals of type I_L. Hence those individuals in class S_L or I_L have been infected L or more times.

We note that this improved pairwise model will only be of benefit during the early growth phase of an outbreak; at equilibrium we will inevitable reach the situation where all individuals have been infected at least L times and hence the equilibrium prevalence predicted by this model and the standard pairwise model must be identical. Somewhat surprisingly, though, the endemic equilibrium of the pairwise model is approached well before the reinfection counting upper limit L is reached. An intuitive explanation for why this is the case can be obtained by considering that in order to reach endemicity, triples of the kind $[I_p S_0 I_q]$ must become prevalent where the I_p and I_q were first infected by a route that did not involve the central S, i.e. when the infection is about to invade completely a loop in the network (no matter how large). In other words, the reinfection counting does not address the inability of the pairwise model of capturing the impact of loops in the network. Reinfection counting can accurately adjust for the case where such a triple was (for example) created by the events $[ISS] \to [IIS] \to [III] \to [ISI]$, but not the case where the central individual has only ever been susceptible.

Computation of system's dimension

We have noted that as k, m or n becomes large, the dimension of the system, and hence the number of equations needed to capture its behaviour becomes large. Here we show how to compute the number of distinct possible states of a neighbourhood (and hence the dimension) using a recursive approach.

For the neighbourhood model, each neighbourhood consists of a central node and k branches. Denote by n_S, B_n and N_n the numbers of distinct states of a single node, the dimension needed to capture a branch of an n-neighbourhood and the dimension of the entire n-neighbourhood, respectively. For a regular graph, $N_1 = n_S$ and $B_2 = n_S$, and for the SIS model on a regular graph $n_S = 2$.

For a k-regular network, the central node of an n-neighbourhood can be in any of the n_S states, and for each of its state, each of the k branches can be in any of the B_n states, so that:

$$N_n = n_S \binom{k + B_n - 1}{k} - 1 \qquad n = 2, 3, \ldots \tag{22}$$

The '-1' comes from the fact that probabilities in being in each possible state sum to 1. The binomial coefficient in (Eq 22) comes from a combinatorial argument, considering the number of ways branches can be arranged around the central node (accounting for symmetries).

The value of B_n can be computed in a similar fashion, as the branch of a n-neighbourhood consists of a root node (attached to the central node of the neighbourhood), which can be in any of the n_S states, and $k-1$ sub-branches. Therefore:

$$B_n = n_S \binom{k - 1 + B_{n-1} - 1}{k - 1} \qquad n = 2, 3, \ldots \tag{23}$$

(Eq 22) and (Eq 23) take into account symmetries to reduce the dimensionality of the system. However, if equations are generated automatically, it might be simpler to number each branch and maintain the order of the branches, resulting in

$$\widetilde{N_n} = n_S B_n^k \tag{24}$$

equations.

The approach described above applies to the neighbourhood model, and therefore covers also the cases of $k = 2$ when the number of nodes m is odd. Alternative, for any m, the number of possible combinations is $n_S^m = 2^m$, but the dimension of the equations can be reduced by considering symmetries within the system which mean that some combinations are effectively counted twice. Consider the odd and even m cases separately. When m is odd, the configuration is symmetric when the left-hand $(m-1)/2$ nodes are the mirror image of those on the right, whatever the state of the central node; hence there are $2 \times 2^{(m-1)/2}$ symmetric combinations. So non-symmetric cases (of which there are $2^m - 2^{(m+1)/2}$) are counted twice (themselves and their mirror-images); therefore, for m odd the dimension of the system is:

$$\frac{1}{2}\left[2^m - 2^{(m+1)/2}\right] + 2^{(m+1)/2} - 1 \; = \; 2^{m-1} + 2^{(m-1)/2} - 1 \; .$$

Similarly, when m is even, the number of symmetric combinations is $2^{m/2}$, and hence the dimension of the system becomes:

$$\frac{1}{2}\left[2^m - 2^{m/2}\right] + 2^{m/2} - 1 \; = \; 2^{m-1} + 2^{(m/2)-1} - 1 \; .$$

Finally, in the case of motif approximation, the system's dimension is more cumbersome to express in general as m becomes large, as it depends on both the number of possible motifs and particular configuration of each one.

Methods for numerical simulation

Although numerical simulation of network-base dynamics is relatively straightforward using standard Gillespie-type algorithms, a range of computational methods can be used to increase the accuracy of any simulation measures. In particular, we wish to use numerical simulations of SIS dynamics on simple degree-3 networks to determine the early growth rate in the amount of infection and the mean prevalence of infection. For both of these we utilise the fact that the equations like (Eq 5) or (Eq 3) provides the exact expected rate of change when the number of pairs or triples (or neighbourhoods) is measured directly from the network simulations.

When calculating the early growth rate, it is possible to use an idealised network. In particular we use a Cayley tree, where a central node is connected to $k = 3$ others, and each successive node is in turn connected to $k - 1 = 2$ new nodes to produce an ever branching network. This network is truncated at 'leaves' which are a fixed distance from the central node. The advantage of this network is that it has no loops, and so it conforms with the assumption in the approximation models given above. Infections are started at the central node, network simulations run until infection hits an outer leaf and the density of pairs and singles measured over time (denoted as $[.]_N(t, e)$ for numerical simulations at time t and replicate simulated epidemic e). Simulations that stochastically fail to reach an outer leaf are ignored.

After an early phase where the number of infected individuals is small and hence stochastic behaviour dominates, we expect to observed exponential growth at rate r in the density of infected cases over time, $[I]_N \sim \exp(rt)$. However, fitting to such exponential growth is made difficult by stochastic delays in the early stages leading to individual simulations having different temporal lags, so the naïve approach of simply averaging over multiple simulations performs poorly. A much simpler alternative is to return to the underlying ODEs (Eq 3) and fit to the expected rate of change:

$$\mathbb{E}\left[\frac{\mathrm{d}[I]_N(t,e)}{\mathrm{d}t}\right] \equiv \tau[SI]_N(t,e) - \gamma[I]_N(t,e) \sim r[I]_N(t,e).$$

The early growth rate r can then be determined by simple linear regression using all replicates and all time points. Identifying r is this manner provides far more accuracy for a given number of simulations than attempting to fit exponential growth curves. We perform this numerical method for multiple transmission rates and hence derive r as a function of τ (assuming $\gamma = 1$).

For the endemic level of infection we take a related approach. We are no longer able to utilise a Cayley tree, as the lower number of connections associated with leaves would affect the dynamics. Instead we generate large networks using the Molloy-Reed (or configuration) algorithm [48], but ensure that self connections, multiple connections between nodes and short loops (of five or less connections) are removed by randomly shuffling connections. In theory, the presence of longer loops will impact on the dynamics, but by examining ever larger networks (where average loops are progressively longer) we believe that there is negligible effect of loops on the mean prevalence obtained from our simulations. The precise numerical value of the expected prevalence is again found by examining at the expected rates of change; close to the expected value $\mathbb{E}[I]$, the rate of

change is expected to be of the following form:

$$\begin{aligned}\mathbb{E}\left[\frac{\mathrm{d}[I]_N}{\mathrm{d}t}\right] &\equiv \tau[SI]_N - \gamma[I]_N \\ &\sim -r_1([I]_N - \mathbb{E}(I)) + r_2([I]_N - \mathbb{E}(I))^2 + \text{h.o.t.} \\ &\sim r_2[I]_N^2 - (2r_2\mathbb{E}(I) + r_1)\,[I]_N + \left(r_1\mathbb{E}(I) + r_2\mathbb{E}(I)^2\right),\end{aligned}$$

where we have dropped the explicit dependence on time and epidemic simulation number. Here the three parameters (r_1, r_2 and $\mathbb{E}[I]$) can be determined by matching this quadratic form to the expected rate ($\tau[SI]_N - \gamma[I]_N$). Figure 2 illustrates the benefits this numerical procedure against simply averaging the values of $[I]_N$ from simulations (assuming $\mathbb{E}[I] = \overline{I_N}$). When conducted using many simulations (or long time frame) both methods agree on the numerical value of the prevalence; however, using the expected rate of change (Eq 25) substantially reduces the between simulation variation and therefore leads to more rapid convergence on the true value. We calculate the expected prevalence $\mathbb{E}[I]$ as a function of the transmission rate τ.
